# An across breed, diet and tissue analysis reveals the transcription factor *NR1H3* as a key mediator of residual feed intake in beef cattle

**DOI:** 10.1186/s12864-024-10151-2

**Published:** 2024-03-04

**Authors:** Kate Keogh, D. A. Kenny, P. A. Alexandre, M. McGee, A. Reverter

**Affiliations:** 1 Animal and Bioscience Research Department, Teagasc, Animal & Grassland Research and Innovation Centre, Grange, Dunsany, Co. Meath Ireland; 2 Queensland Bioscience Precinct, CSIRO Agriculture & Food, 306 Carmody Rd., St. Lucia, 4067 Brisbane, QLD Australia; 3 Livestock Systems Research Department, Teagasc, Animal & Grassland Research and Innovation Centre, Grange, Dunsany, Co. Meath Ireland

**Keywords:** Feed efficiency, Beef cattle, Gene co-expression network analysis

## Abstract

**Background:**

Provision of feed is a major determinant of overall profitability in beef production systems, accounting for up to 75% of the variable costs. Thus, improving cattle feed efficiency, by way of determining the underlying genomic control and subsequently selecting for feed efficient cattle, provides a method through which feed input costs may be reduced. The objective of this study was to undertake gene co-expression network analysis using RNA-Sequence data generated from *Longissimus dorsi* and liver tissue samples collected from steers of two contrasting breeds (Charolais and Holstein-Friesian) divergent for residual feed intake (RFI), across two consecutive distinct dietary phases (zero-grazed grass and high-concentrate). Categories including differentially expressed genes (DEGs) based on the contrasts of RFI phenotype, breed and dietary source, as well as key transcription factors and proteins secreted in plasma were utilised as nodes of the gene co-expression network.

**Results:**

Of the 2,929 DEGs within the network analysis, 1,604 were reported to have statistically significant correlations (≥ 0.80), resulting in a total of 43,876 significant connections between genes. Pathway analysis of clusters of co-expressed genes revealed enrichment of processes related to lipid metabolism (fatty acid biosynthesis, fatty acid β-oxidation, cholesterol biosynthesis), immune function, (complement cascade, coagulation system, acute phase response signalling), and energy production (oxidative phosphorylation, mitochondrial L-carnitine shuttle pathway) based on genes related to RFI, breed and dietary source contrasts.

**Conclusions:**

Although similar biological processes were evident across the three factors examined, no one gene node was evident across RFI, breed and diet contrasts in both liver and muscle tissues. However within the liver tissue, the *IRX4*, *NR1H3*, *HOXA13* and *ZNF648* gene nodes, which all encode transcription factors displayed significant connections across the RFI, diet and breed comparisons, indicating a role for these transcription factors towards the RFI phenotype irrespective of diet and breed. Moreover, the *NR1H3* gene encodes a protein secreted into plasma from the hepatocytes of the liver, highlighting the potential for this gene to be explored as a robust biomarker for the RFI trait in beef cattle.

**Supplementary Information:**

The online version contains supplementary material available at 10.1186/s12864-024-10151-2.

## Background

There is increasing pressure on the global agri-food industry to reduce its environmental footprint, while simultaneously meeting the growing demand for animal protein [1]. Within beef production systems, feed provision is a major determinant of overall profitability as it accounts for up to 75% of the total variable costs of production [2]. Consequently, the identification and subsequent breeding of beef cattle with improved feed efficiency has received attention as a means to alleviate the high input costs, as well as enhancing the environmental sustainability of beef production [2, 3]. In beef cattle breeding selection programmes, residual feed intake (RFI), defined as the difference between an animal’s actual and predicted feed intake, has become the feed efficiency measure of choice due to its moderate heritability (0.26–0.54) and independence from production traits [4, 5].

However, despite the clear benefits of breeding cattle that are more feed efficient, the measurement of RFI in cattle requires an expensive and often labour-intensive performance measurement period during which individual animal feed intake and weight gain are recorded usually over at least 70 days [2]. This has led to considerable research efforts aimed towards the discovery of accurate biological markers for RFI [2, 5]. Nonetheless, despite recent research efforts aimed at uncovering the molecular control governing RFI in cattle, key robust genes or genomic regions contributing to the trait are yet to be identified [2]. This is undoubtedly due to the multifaceted nature of the RFI trait coupled with variation in experimental factors across different studies, such as differences in animal breed, developmental stage, and diet, which ultimately confound the outcomes. Moreover, the potential for a genotype × environment interaction manifested as inconsistent feed efficiency and animal performance ranking across different diet types is an additional concern for forage-based beef production systems, such as that practiced in Ireland and many temperate regions throughout the world. Additionally, it is well established that different breeds have different inherent feed efficiency capacity [2]. Furthermore, although relatively repeatable, re-ranking of cattle for RFI when offered either the same diet [6] or diets differing in chemical composition/energy density [7] over successive feed intake recording periods has been observed. In light of this, research to elucidate the genotype × environment interaction in relation to RFI in beef cattle is warranted in order to inform future breeding programs.

Thus, the objective of this study was to undertake gene co-expression network analysis using global transcriptomics data generated from *Longissimus dorsi* - an economically important and metabolically active muscle, associated with RFI [5] - and liver tissue - a major metabolic organ associated with RFI [8] derived from steers of two contrasting breed types (Charolais and Holstein-Friesian) divergent, within breed, for RFI across consecutive contrasting dietary phases. Specifically, we sought to identify co-expressed genes that were commonly associated with and contributing to the RFI trait across the two contrasting breeds utilised and the varying diets offered. Gene co-expression network approaches are based on determining the interaction between genes, such that a change in the expression of one gene may be propagated through interactions and affect the expression of other genes [9]. Furthermore, the application of co-expression network analyses to transcriptomic datasets may allow for the identification of more complex transcriptional regulation which may not be detectable through differential expression analysis alone. Thus, applying co-expression network analysis to transcriptomic datasets of cattle divergent for RFI across various breed types and offered contrasting diets may reveal more insights into the underlying biology governing the effect of genotype and dietary source on RFI phenotype in beef cattle.

## Results

### RFI phenotype classification

Descriptive results pertaining to growth, dietary intake and RFI values are outlined in full in Higgins et al. [10]. High-RFI steers consumed more feed on average than their Low-RFI counterparts (*P* < 0.001), while having a similar ADG (*P* > 0.05). Specifically, High-RFI Charolais steers consumed 16%, 8%, and 15% more than their Low-RFI contemporaries for the H1, ZG and H2 diets, respectively. Similarly, for the Holstein-Friesian population, High-RFI steers consumed 12%, 11% and 17% more feed than their Low-RFI contrasts for H1, ZG and H2 diets, respectively.

### Differential expression analysis

**Fig. 1:**
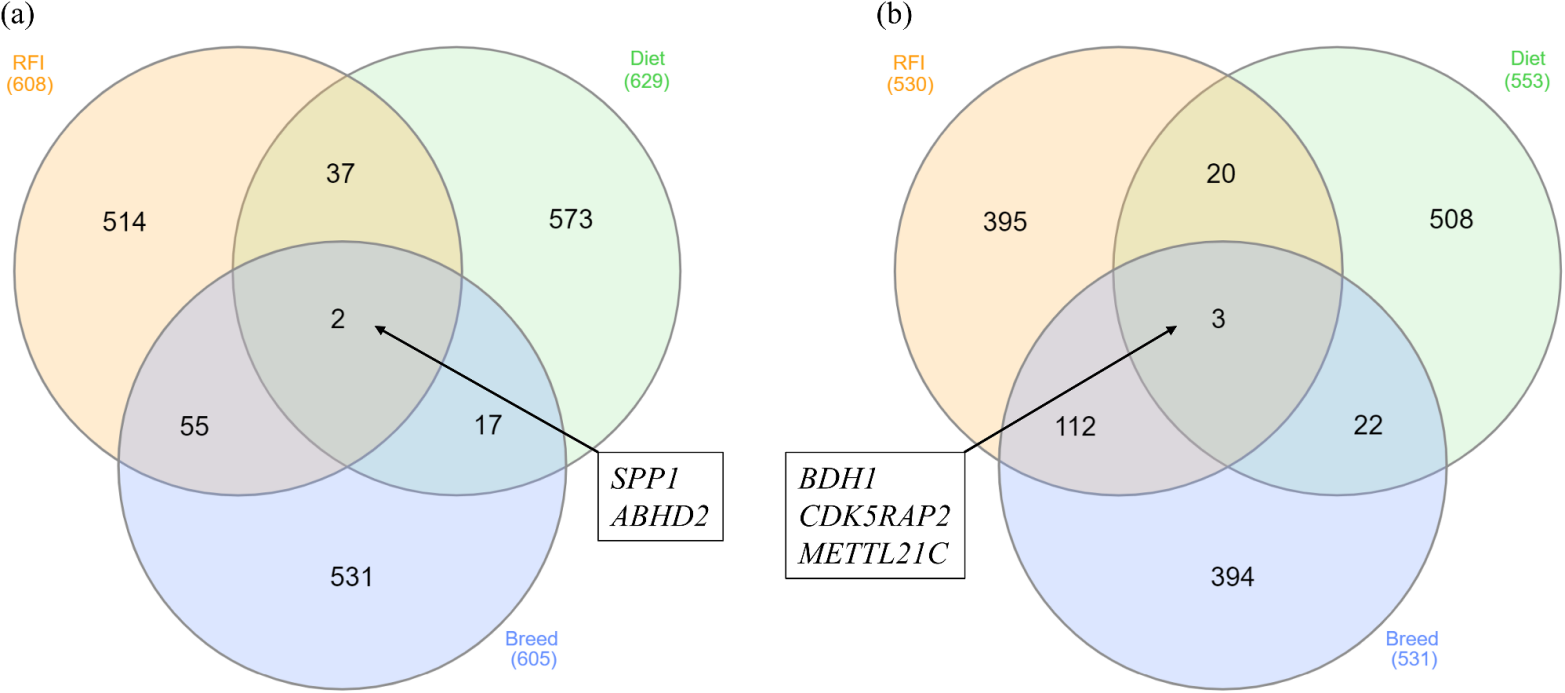
Venn diagrams showing DEGs for each RFI, diet and breed contrast in (**a**) liver and (**b**) muscle. Within the liver tissue two genes, *SPP1* and *ABHD2* were differentially expressed across RFI, breed and diet contrasts, whilst *BDH1*, *CDK5RAP2* and *METTL21C* were differentially expressed across the three contrasts tested in the skeletal muscle tissue

From a total of 27,607 genes present within the bovine reference genome (ARS1.2-UCD), 13,304 genes (48.2%) were identified as being expressed in at least one of the six contrasts undertaken, following quality control procedures. Numbers of genes retained in each of the six contrasts following filtering for lowly expressed genes are presented in Table 1, along with the numbers of genes differentially expressed, based on the top 5% differentially expressed following correction for multiple testing. The identity of the top 5% DEGs for each contrast is provided in full in Additional Table 1. Of the genes designated as differentially expressed within this study, no gene was commonly reported as differentially expressed across the six contrasts evaluated. However, when examining each distinct tissue, we observed the highest percentage of common genes in the breed contrast, followed by the RFI contrast. Conversely, the diet contrast revealed the fewest common genes in both the liver and muscle (Fig. 1). Within each tissue, 2 and 3 genes were identified as differentially expressed across the RFI, diet and breed contrasts examined in liver and muscle tissues, respectively. Within the liver tissue, these were the *SPP1* and *ABHD2* genes, whilst *BDH1*, *CDK5RAP2* and *METTL21C* were present across the RFI, breed and diet contrasts within the muscle tissue.

**Table 1:** Numbers of genes expressed and identified as differentially expressed within RFI, diet and breed contrasts across liver and muscle tissues

Contrast	Number of expressed genes	Top 5% DEG^1^	Genes specific to contrast^2^	Genes in common with other contrasts
Liver-RFI	12,161	608	454 (74.7%)	25.3%
Liver-diet	12,581	629	511 (81.2%)	18.8%
Liver-breed	12,114	605	421 (69.6%)	30.4%
Muscle-RFI	10,618	530	333 (62.8%)	37.2%
Muscle-diet	11,075	553	424 (76.7%)	23.3%
Muscle-breed	10,655	531	312 (58.8%)	41.2%

^1^All expressed genes were assessed for differential expression and the top 5% most significantly different of the expressed genes were deemed to be DEGs

^2^Considering all six contrasts

### Co-expression network analysis

**Fig. 2:**
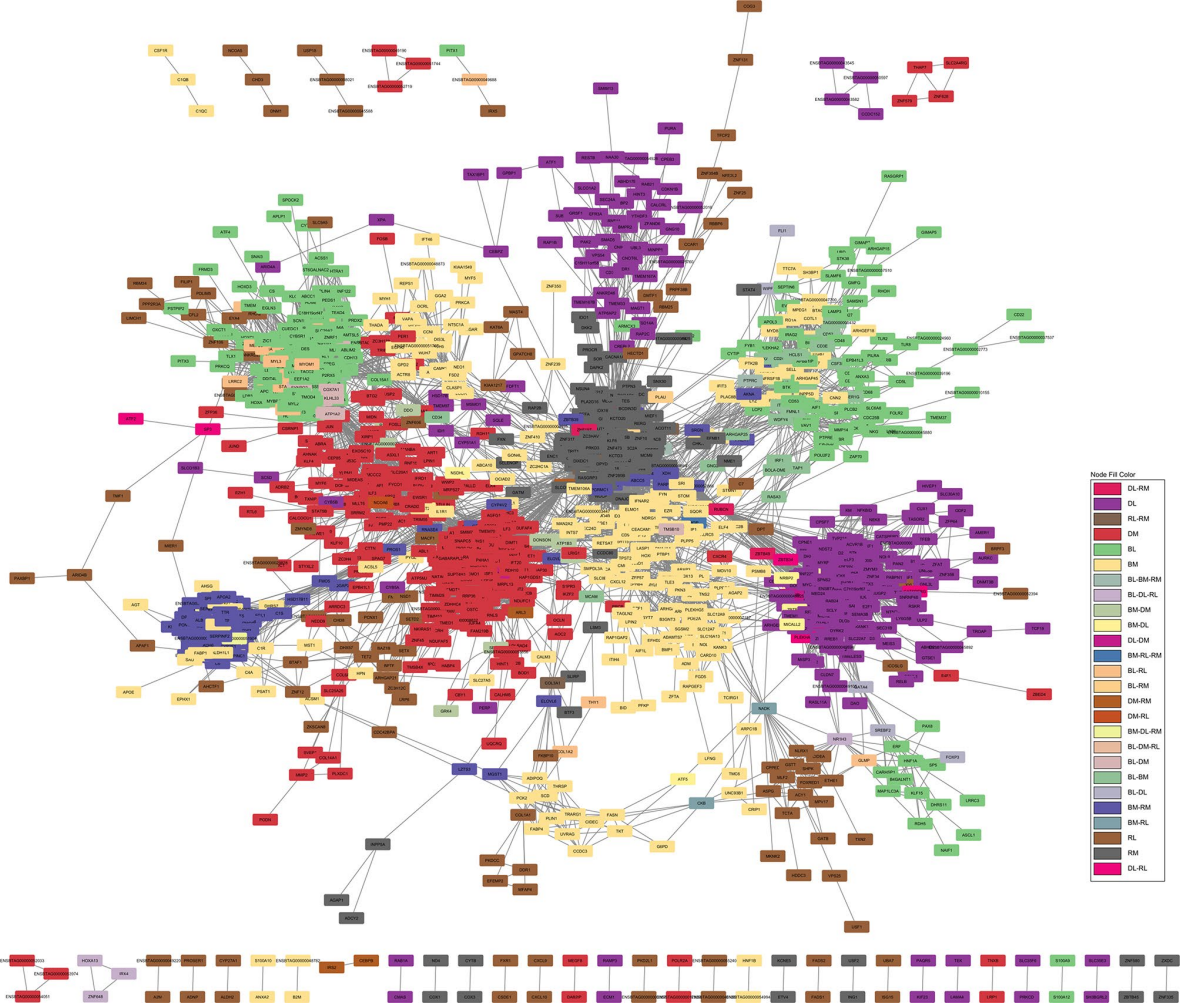
Gene co-expression network constructed using PCIT algorithm on 1,604 selected genes displaying significant correlations (≥ 0.8), with a total of 43,876 connections presented. Gene nodes were based on DEGs pertaining to contrasts of High- versus Low-RFI, Charolais versus Holstein-Friesian and HC diet versus ZG diet. Legend: RL = RFI in liver contrast; RM = RFI in muscle contrast; DL = diet contrast in liver; DM = diet contrast in muscle; BL = breed contrast in liver and; BM = breed contrast in muscle tissue

**Fig. 3:**
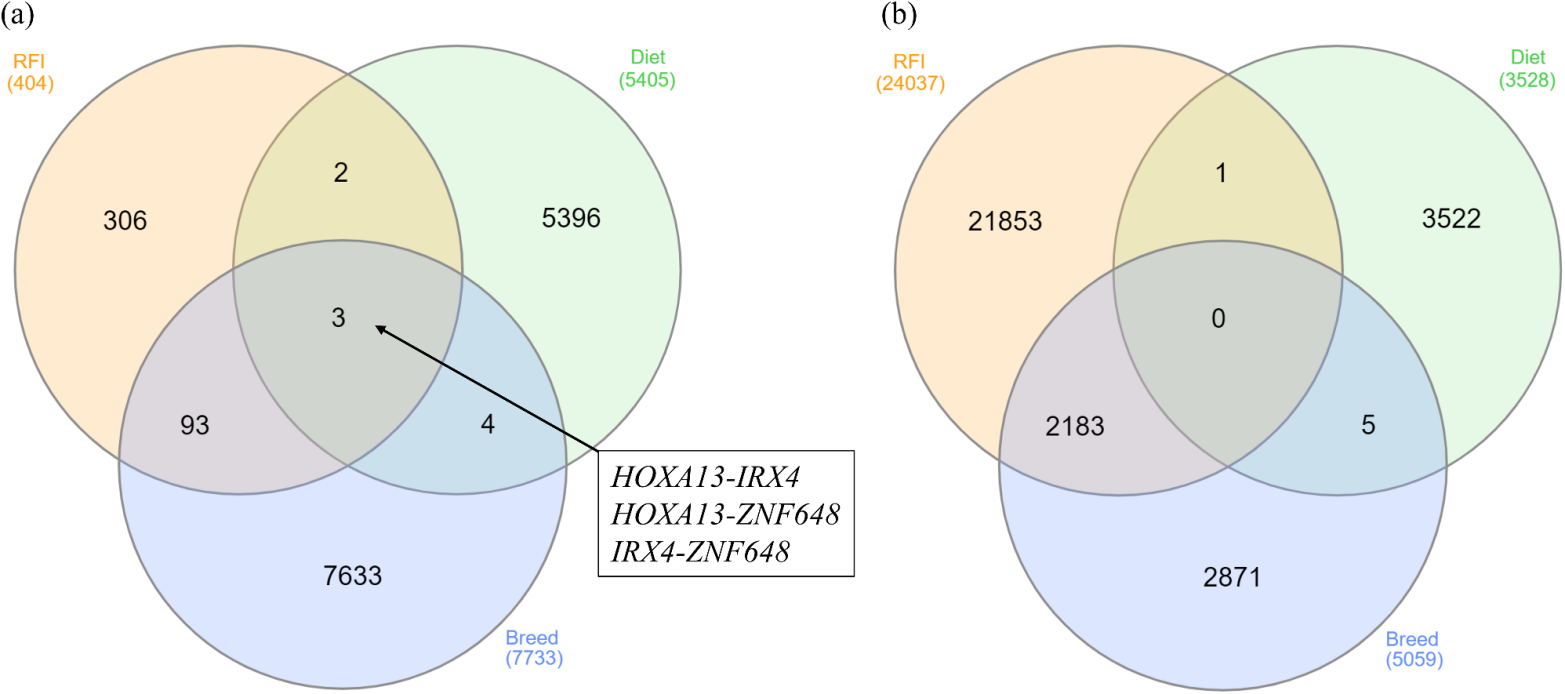
Venn diagrams showing significant connections for each RFI, diet and breed contrast in (**a**) liver and (**b**) muscle. Within the liver tissue three connections were common across the RFI, breed and diet contrasts examined, whilst no commonality was detected within the skeletal muscle tissue

**Fig. 4:**
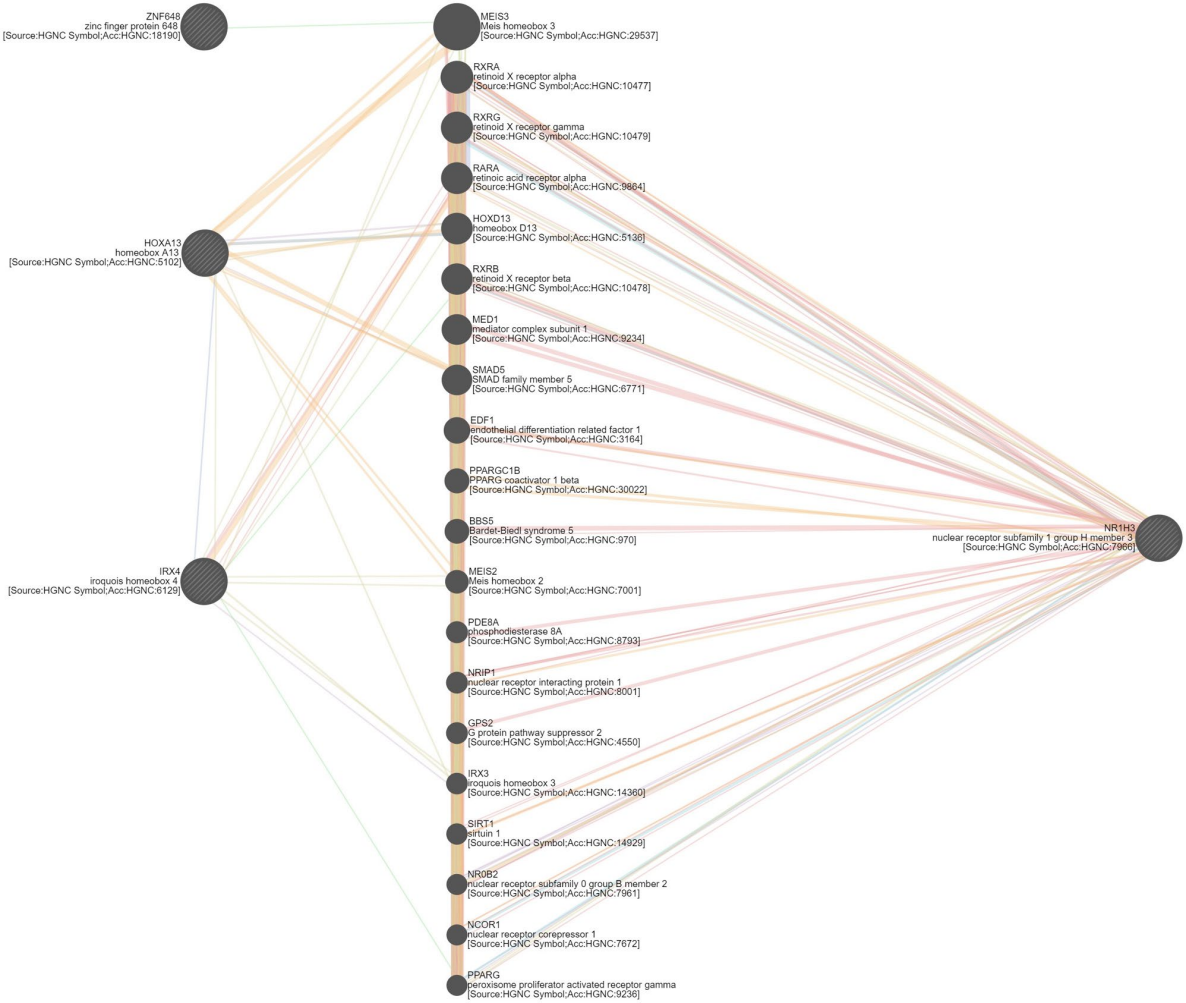
Biological interactions of the four genes (*HOXA13*, *IRX3*, *NR1H3* and *ZNF648*) identified within the RFI, breed and diet contrasts within the liver tissue. The interactions between the four genes examined were retrieved from the GeneMANIA database [29]

Of the 2,929 DEGs used as input nodes within the network analysis, 1,604 were reported to have significant correlations, resulting in a total of 43,876 significant connections between genes (correlation ≥ 0.80). The resultant co-expression network for the contrasts of RFI, breed and diet for both liver and skeletal muscle tissues is presented in Fig. 2 (also presented in Additional Fig. 1, without overlap of gene nodes). Of the 1,604 nodes with significant connections, 215 or 7.6% were known bovine transcription factors, whilst 152 or 7.6% represented genes encoding proteins secreted from either skeletal muscle or liver tissue into the plasma. The 43,876 significant connections were comprised of 7,733 within the breed contrast in the liver tissue; 5,504 in the diet contrast in the liver; 404 in the RFI contrast in the liver; 5,059 in the breed contrast in muscle tissue; 3,528 in the diet contrast in muscle and 24,037 connections in the RFI contrast in the muscle tissue. Numbers of gene nodes for each contrast are outlined in Table 2, with commonality between the various contrasts examined presented in Fig. 3. Of the 1,604 gene nodes with significant connections no single gene was considered significant across all six differential expression contrasts utilised for network analysis. However, within the liver contrasts, four genes, namely *IRX4*, *NR1H3*, *HOXA13* and *ZNF648* displayed significant connections across the RFI, diet and breed comparisons. The biological functions of these four genes were evaluated through GeneMANIA [11], resulting in the identification of genes biologically connected to the four genes examined (Fig. 4). Through Cytoscape software, the MCODE application was utilised to determine clusters of highly interconnected genes. In total 12 clusters of highly interconnected genes were identified through the MCODE function, these clusters are highlighted within Additional Fig. 2. Moreover, biological pathways enriched within each cluster, determined through IPA are outlined in Additional Table 2. Enriched pathways within the diet, breed and RFI network included those related to lipid metabolism (triacylglycerol biosynthesis, fatty acid biosynthesis, fatty acid β-oxidation, cholesterol biosynthesis), immune function, (complement system, coagulation system, acute phase response signalling), energy production (oxidative phosphorylation, mitochondrial L-carnitine shuttle pathway), and cellular growth (growth hormone signalling, cyclin and cell cycle regulation).

**Table 2:** Details of genes pertaining to each contrast identified with significant connections and included within the co-expression network in Fig. 2

Contrast	Connections	Genes	TF	Secretome	Genes specific to contrast
Liver-RFI	404	161	30	14	117 (72.6%)
Liver-diet	5405	299	70	4	263 (87.9%)
Liver-breed	7733	312	44	45	237 (75.9%)
Muscle-RFI	24,037	392	35	68	267 (68.1%)
Muscle-diet	3528	301	40	7	273 (90.6%)
Muscle-breed	5059	375	35	80	226 (60.2%)

TF = transcription factor; Secretome = gene encoding proteins secreted in plasma from liver and muscle tissues

**Fig. 5:**
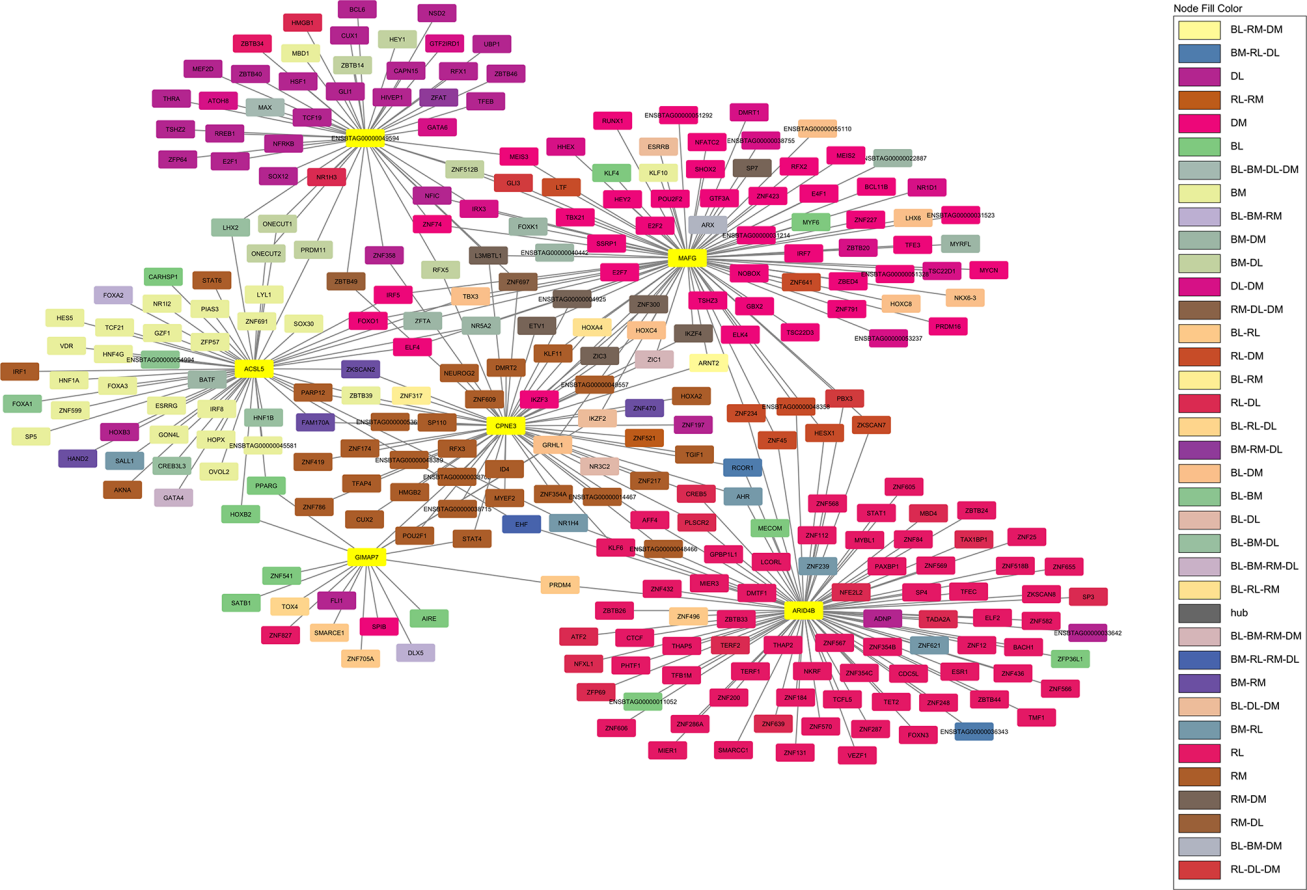
Network depicting the interaction between the most interconnected genes within each of the six contrasts of diet, RFI and breed across liver and muscle tissues undertaken. Most interconnected genes are highlighted in yellow and include *ARID4B* in the liver-RFI contrast; *CPNE3* in the muscle-RFI contrast; *ENSBTAG00000049594* in the liver-diet contrast; *MAFG* in the muscle-diet contrast and; *GIMAP7* and *ACSL5* in liver and muscle, respectively for the breed contrasts. Legend: RL = RFI in liver contrast; ML = RFI in muscle contrast; DL = diet contrast in liver; DM = diet contrast in muscle; BL = breed contrast in liver and; BM = breed contrast in muscle tissue

Within each of the six diet, RFI, and breed contrasts for each tissue, the most interconnected genes were identified and the first neighbours of these gene nodes that are transcription factors determined. For the RFI contrasts, *ARID4B* was the most interconnected gene node in the liver dataset with 256 connections, whilst with 415 connections, *CPNE3* was the most interconnected gene node within the muscle-RFI contrast. In the liver-diet contrast, *ENSBTAG00000049594* was the most interconnected gene with 333 connections; *MAFG* was the most connected gene node in the diet contrast from the muscle tissue dataset with 467 connections. *GIMAP7* and *ACSL5* were the most interconnected genes in the breed contrasts for liver and muscle tissues, with 517 and 413 connections, respectively. The connections for these specific genes represented 63.3, 6.2, 6.7, 1.7, 13.2 and 8.2% of all the significant connections for the liver-RFI, liver-diet, liver-breed, muscle-RFI, muscle-diet and muscle-breed contrasts, respectively. A total of 40, 62, 77, 86, 99 and 54 transcription factors were identified as first neighbours for *GIMAP7*, *ACSL5*, *ENSBTAG00000049594*, *MAFG*, *ARID4B* and *CPNE3*, respectively. First degree transcription factor neighbours of the most interconnected genes were evident across multiple diet, breed and RFI contrasts examined in this study. This is evident within Fig. 5, which depicts a network presenting the interaction between the most interconnected genes and their first neighbours which are known transcription factors in the bovine genome.

## Discussion

Results from this study identify co-expressed genes related to variation in RFI, as well as contrasting breed and dietary source. Although results from the current study highlight interactions amongst genes governing variation in RFI, breed and dietary source, the use of the same animals across each of these contrasts represents a potential limitation. Overall, nearly two thirds of the animals used in this study were included in more than one of the various contrasts examined (tissue, breed, RFI and dietary source; Additional Table 3), thus highlighting a potential imbalance of animals contributing to each contrast and consequently presenting a potential caveat of the results generated, which must be considered in terms of the subsequent interpretation. However, despite multiple animals being used across the contrasts examined, no common gene was reported across the RFI, breed and diet type contrasts in both liver and muscle tissues, suggesting the use of the same animals across multiple contrasts did not bias the results produced. Notwithstanding the lack of common genes across all contrasts, similar biological processes were affected across the RFI, breed and diet contrasts, namely lipid metabolism, immune response and energy production. Thus the remainder of thisdiscussion will focus on these important biological functions.

### Metabolism and lipid biosynthesis

Variation in metabolism related processes has been reported as implicated towards the RFI phenotype within the published literature [5], indeed, a similar finding was observed in the current study. For example, a cluster of co-expressed genes pertaining to the RFI contrast within the liver tissue contained genes involved in metabolism and lipid biosynthetic processes including *ASPG*, *CIDEA*, and *CPPED1*. The inclusion of *CIDEA* within the RFI contrast in the liver was of particular relevance due to the role of the CIDE proteins towards metabolic rate, which has been attributed to be different between cattle divergent for RFI [2, 5]. Moreover, another member of the CIDE family of proteins, *CIDEC*, was identified as co-expressed within a separate cluster of co-expressed genes related to the breed contrast in the muscle tissue. This result is of interest as although CIDEC was not reported to be related to RFI in the current network based analysis, the *CIDEC* gene has previously been implicated towards variation in RFI. Specifically, *CIDEC* was reported as down-regulated in skeletal muscle tissue of Low-RFI Holstein-Friesian and Charolais steers across various diets [12] and in liver tissue of Low-RFI Charolais steers following a grass diet [10]. Through the gene network generated in this study, *CIDEC* was interacting with other genes with functions in lipid metabolism, including for example *TRARG1*, which similar to *CIDEC* was down-regulated in skeletal muscle of Low-RFI cattle across varying breeds and dietary sources in Keogh et al. [12]. *CIDEC* and *TRARG1* are both regulated by insulin, with the latter of these genes involved in glucose import in response to insulin stimulus. Insulin is known to play important roles in both lipogenesis and lipolysis, thus, the inclusion of insulin responsive genes within a cluster of co-expressed genes related to breed variation in muscle tissue may indicate a role for these genes towards variation in lipid metabolism or fatty acid content in the *longissimus dorsi* muscle of Charolais and Holstein-Friesian cattle. Indeed, intramuscular fat content and fatty acid composition are affected by cattle breed [13], which may be attributable to differences in glucose processing and insulin signalling in muscle tissue. Moreover, both genes were previously reported as differentially expressed between cattle divergent for feed efficiency phenotype, potentially suggesting a role for these genes towards RFI across varying breed type. Two other genes of interest in this cluster were *FASN* and *SCD*, which both contribute to fatty acid synthesis, and both of which were previously reported as differentially expressed in cattle divergent for feed efficiency phenotype. For example, *FASN* was differentially expressed between cattle divergent for RFI in skeletal muscle of Charolais steers following a high-concentrate diet [12]; in liver tissue of Nellore steers [14] and in adipose of Angus bulls [15]. Whilst *SCD* was differentially expressed in Charolais steers following a zero-grazed grass diet [12] as well as in adipose in Angus bulls [15]. Moreover, in the study of Mukiibi et al. [16] where genes differentially expressed between cattle divergent for RFI were detected across three separate breed types, *SCD* was reported as one of only five genes commonly differentially expressed across all three breeds examined. Thus, the presence of these genes as differentially expressed within the literature highlights a role for these two genes towards RFI, but also towards variation in skeletal muscle metabolism and fatty acid content between the breed types examined in the current study.

Additionally, a separate cluster of co-expressed genes primarily comprised of muscle specific genes for both breed and RFI contrasts, contained genes enriched for biological processes related to synthesis of cholesterol and other lipids. Indeed, genes within this cluster were previously reported as differentially expressed between cattle divergent for RFI, including *CYP1A2* [17], *CYP2C19* [16], *CYP2E1* [14, 15, 18], *EPHX1* [10] and; *FABP1* [15, 19]. Interestingly, *APOE* which encodes an apolipoprotein was also present in this cluster, specifically through its inclusion in the breed comparison. *APOE* is involved in the delivery of triglycerides and energy storage in muscle, highlighting differences in the fatty acid content and muscle energy storage capacity of the same muscle between the two breeds examined. Although not pertaining to the RFI contrast, variation in the *APOE* gene between breeds may also contribute to variation in skeletal muscle energy metabolism, due to differences in energy storage processes which may contribute to RFI variation. Moreover, a separate cluster of co-expressed genes pertaining to the liver-breed contrast included genes involved in glycolytic processes and glycogen synthesis, which may also suggest differing hepatic energy storage capacities between the two breeds used. Thus, the effect of both RFI and breed on the interaction between these genes may be a direct consequence of the amount or type of energy consumed as well as variation in the metabolic processing in the skeletal muscle and liver between the breeds divergent for RFI. Similarly, a separate cluster of co-expressed genes also pertaining to the breed contrast in muscle tissue, also displayed enrichment for fatty acid synthesis and metabolism processes. Genes related to fatty acid metabolism in this cluster included *DGAT1*, *LCAT, LPIN2*, *MLXIPL* and *RETSAT*. Additionally, *ELOVL5* and *ELOVL6* which are both involved in the elongation of fatty acids were also present in this cluster, both of these genes have been reported as differentially expressed between cattle divergent for RFI [12, 15, 16, 20]. Moreover, these two genes were also present within the RFI contrast in the current study indicating a potential role for these genes towards mediating the interaction between RFI and breed type. Furthermore, the *ACSL5*, gene, involved in lipid metabolism and identified as the most interconnected gene within the muscle-breed contrast was located between one cluster of co-expressed genes related to the muscle-breed contrast and another cluster related to the muscle-diet contrast. First neighbours of the *ACSL5* gene within the transcription factor network included those involved in lipid related metabolism including *CREBL2* and *PPARG*, both of which were differentially expressed between cattle divergent for RFI in Weber et al. [15]. Moreover, the relationship between insulin and glucose with lipid metabolism is further highlighted through the interaction of *ACSL5* with *FOXO1* and *ONECUT1* which are involved in insulin signalling and glucose metabolism, respectively, indeed both were previously differentially expressed between cattle divergent for RFI [10, 12]. Together these results highlight a potential role for *ACSL5* towards variation in skeletal muscle of varying breeds, which may be dependent on the diet type offered and consequent glucoregulatory processes. Thus, whilst not all of the aforementioned genes were identified through the RFI contrast in the current co-expression study, the identification of the same genes across other studies in the literature, highlights a potential role for these genes towards variation in RFI irrespective of breed type. Moreover, the identification of genes involved in lipid metabolism in the aforementioned clusters, which were specific to the muscle breed contrast, highlights the differential lipid metabolism of the longissimus muscle between the two contrasting breeds examined and potentially differing energy consumption between the two breeds in both tissues examined.

### Immune response

Divergence in RFI has been attributed to differences in immune response [10, 16]. Indeed, a similar result was evident in a cluster related to both RFI and breed contrasts within the muscle tissue. Pathway analysis of this cluster revealed enrichment of pathways related to the complement system and coagulation cascade. Moreover, this cluster was also enriched for lipid metabolism as discussed above. Thus, genes within this cluster are at the intersection of the two main processes attributed to RFI within the published literature, moreover these genes were included within both breed and RFI contrasts in muscle, representing potential markers for RFI irrespective of breed type examined. Indeed, the role for genes functioning in these processes towards RFI as well as across varying breed and management type is evident through the prior identification of genes involved in coagulation and complement cascade within the published literature. For example, genes of the complement system, which is involved in mediating the innate immune response were previously reported as differentially expressed between cattle divergent for RFI including *C1R, C2, C1S, C4A, C3, C5, C9, CFI* and *CRP* [10, 12, 15, 21]. Additionally, other genes of the complement system were also included within this cluster, *C6, C8A, C9* and *CFB*, highlighting additional genes of the complement system not previously identified in relation to RFI, but potentially important to the underlying biology governing the expression of the trait. Similarly in the current study, genes involved in the coagulation cascade were co-expressed including *F2, PLG, SERPIND1, F5, FGA, FGB, FGG, FGL1, SERPINC1, SERPINF2, KNG1* and *PLG*. Moreover, a number of these genes were previously reported as differentially expressed between cattle divergent for feed efficiency phenotype [10, 12, 15, 17, 22]. Indeed, the enrichment of the coagulation system in this cluster may also be due to variation in blood pressure between feed efficient and inefficient cattle as a direct consequence of the quantity of feed consumed. For example, in a study examining the effect of dietary restriction and subsequent re-alimentation in beef cattle, variation in the amount of feed consumed resulted in altered blood pressure responses [23]. Moreover, the network generated in this study included genes related to angiogenesis and vasodilation including *ADM, EPHB4* and *PLEKHG5*, all of which were previously reported as differentially expressed between cattle divergent for feed efficiency phenotype [10, 15, 18], evidencing a potential difference in blood pressure and angiogenesis towards variation in feed efficiency phenotype in cattle.

Whilst the aforementioned immune-based results are specific to the muscle tissue, a separate cluster of co-expressed genes included genes enriched for immune functionality in the liver tissue. However, this cluster was based on the breed contrast and not the RFI contrast in liver tissue. Despite this cluster being based on the breed contrast, genes included within this cluster were previously implicated towards variation in RFI in beef cattle. For example, *SELPLG*, involved in inflammation and *IL2RG,* an interleukin receptor were both differentially expressed in Angus cattle [15] and *F13A1* which encodes a coagulation factor was also differentially expressed in the skeletal muscle of Nellore steers in the data of Tizioto et al. [19]. Interestingly several genes within this cluster pertaining to the liver-breed comparison which had functions related to T cell signalling were previously reported as differentially expressed between cattle divergent for feed efficiency. These included *APBP1IP*, involved in T-cell activation which was differentially expressed in the dataset of Yang et al. [24], both *SPN* and *ZAP70* which encode genes involved in antigen specific activation of T-cells and T-cell development, respectively, and were differentially expressed in the skeletal muscle dataset of Keogh et al. [12], whilst the T-cell receptor, *CD247* was differentially expressed in the dataset of Alexandre et al. [18]. Overall, these results suggest a potential role for T-cell signalling towards both variation in RFI and liver transcriptome between contrasting breed types.

The importance of immune function towards variation in RFI is further established through the transcription factor network analysis undertaken in this study. Specifically, *ARID4B* and *CPNE3* which were identified as the most interconnected genes within liver and muscle RFI contrasts, respectively, were interacting with transcription factors with an associated immune function. These included *NFE2L2, PLSCR2, STAT1* and *TAX1BP1* which were connected to *ARID4B*. *PLSCR2* was of particular interest due to its potential role in blood coagulation, again highlighting a role for coagulation towards variation in RFI phenotype. Similarly, *CPNE3* which encodes a calcium dependent binding protein, which mediates interactions between integrins and extracellular ligands, was identified as the most interconnected gene within the RFI contrast in the muscle tissue. Indeed, from the main RFI, breed and diet network generated in this study, *CPNE3* was included within a cluster of co-expressed genes with associated immune related functions. Through the transcription factor network, *CPNE3* was specifically interacting with genes of the innate immune response (*DDX58*, *ZC3HAV1, RFX5*) and inflammatory response (*CLEC1A*, *CTSS*). Additionally, both *ARID4B* and *CPNE3* were also connected to transcription factors involved in lipid metabolism, suggesting a role for these genes towards mediating the interaction between immune response and lipid metabolism towards variation in RFI.

### Mitochondrial function

In addition to its interaction with genes related to immune processes and lipid metabolism, *CPNE3* was also interacting with genes involved in energy production and mitochondrial processes. A role for differential mitochondrial functioning towards variation in RFI in beef cattle has been suggested previously [2, 5, 25]. Indeed, the results from the current study further highlight a role for mitochondrial functionality towards variation in RFI. For example, *CPNE3*, pertaining to the muscle-RFI contrast, was interacting with genes encoding proteins involved in mitochondrial functionality. Specifically, these included genes of the mitochondrial enzyme complexes (*COX18, NDUFAF7, NDUFS1*), genes involved in CoA biosynthesis (*PANK1, PANK2, PANK3*), a mitochondrial transporter protein (*SLC25A15*); as well as genes involved in mitochondrial regulation (*MIEF1*, *EIF2AK3*, *ERMP1*) and the citric acid cycle (*GPAM*, *IDH1*). Indeed, *SLC25A15, NDUFS1* and *GPAM* were previously reported as differentially expressed in cattle divergent for RFI [15, 16, 25]. Moreover, *IDH1* and *SLC25A15* were also present within the breed contrast in the skeletal muscle tissue, suggesting a role for these genes towards RFI irrespective of breed type examined. Furthermore, a separate cluster of co-expressed genes pertaining to the diet contrast within the muscle tissue was also enriched for oxidative phosphorylation. Additionally, genes included within this diet-specific cluster were recently reported as differentially expressed in the liver tissue of crossbred beef steers divergent for RFI phenotype [25]. These included genes encoding the first and second enzyme complexes of the electron transport chain (*NDUFB5*, *NDUFA5*, *NDUFC1*, *NDUFA4*, *UQCRH*) as well as a gene encoding a mitochondrial amidinotransferase protein (*GATM*). Of these genes, *NDUFA4* was reported as differentially expressed between cattle divergent for feed efficiency in Dorji et al. [22], Higgins et al. [10] and Casal et al. [26], whilst *UQCRH* and *GATM* were differentially expressed in the data of Casal et al. [26] and Mukiibi et al. [16], respectively. Thus, results from this study suggest differential responses of mitochondrial related genes dependent on the contrast examined, which taken together may suggest that these processes contribute to RFI irrespective of dietary source. However, despite the commonality in biological function the same genes were not present between the two clusters, which may indicate towards other regulatory genomic regions contributing to the interaction between dietary source offered and resultant RFI phenotype.

### Key transcription factors regulating RFI

As previously mentioned, through the network analysis conducted in this study, no single gene was identified across both tissues as contributing to RFI irrespective of breed type and diet offered. Notwithstanding this outcome, we did identify four genes as implicated towards RFI, breed and diet within the liver tissue, specifically these included the *IRX4*, *NR1H3*, *HOXA13* and *ZNF648* genes, which all encode transcription factors. Indeed, the commonality of these genes across the three contrasts examined in this study highlights the potential use of these genes as biomarkers for RFI in beef cattle robust across varying breeds and dietary management systems. Moreover, *NR1H3* was of particular interest for further evaluation as a potential biomarker for RFI due to the encoded protein being secreted from hepatocytes of the liver into the plasma. An evaluation of the biological roles of these transcription factors through their known published interactions (Fig. 4) revealed a relationship between these four key transcription factors and biological processes related to steroid hormone receptors (*RXRA, RXRG, RARA, RXRB, MED1, PPARGC1B, SIRT1, NCOR1)*, insulin signalling and glucose homeostasis (*MEIS3, PPARGC1B, GPS2, SIRT1, PPARG*), circadian clock (*NRIP1, SIRT1, NR0B2, NCOR1, PPARG*) and lipid metabolism (*EDF1, GPS2, SIRT1, NCOR1, PPARG*). Indeed, *MEIS2, IRX3, EDF1, RXRG, NR0B2, PPARG* and *NCOR1* have previously been reported as differentially expressed between cattle divergent for feed efficiency potential [15–18, 22, 24], further evidencing a role for these transcription factors towards mediating the RFI phenotype.

Whilst the roles of insulin and lipid metabolism towards RFI have been discussed previously, alterations in circadian clock signalling have been proposed to be contributing to the differential level of feed intake between cattle divergent for RFI [27]. Interestingly the *NR1H3* gene has been shown to display functions related to circadian clock as well as being a transcription factor for lipid metabolism. Additionally, a role for stress physiology towards RFI divergence has also been suggested [2]. Indeed the interaction of the four key transcription factors identified in this study with genes encoding proteins involved in steroid hormone receptor signalling further implicates variation in stress physiology towards RFI phenotype. Moreover, downstream targets of NR1H3 include the glucocorticoid receptor, suggesting a role for this transcription factor in modulating hepatic glucocorticoid action. Indeed, nuclear receptor genes including *NR1H3* have previously been shown to be up-regulated by glucocorticoid hormone administration in adipose tissue [28], whilst glucocorticoids have also been shown to be important downstream regulators of circadian tissue clocks and have essential functions in the physiological adaptation to stress [29]. Moreover, the glucocorticoid receptor has been shown to be a downstream target for SHP inhibition [30], the protein encoded by the *NR0B2* gene. Indeed, within the context of RFI the interaction between *NR1H3* and the orphan nuclear receptor SHP, encoded by *NR0B2* is of particular interest, due to the identification of the NR0B2 gene as consistently down-regulated in liver tissue of Low-RFI cattle [10, 14–16, 18]. Additionally, although it did not pass the correlation strength cut-off for inclusion in the network analysis conducted in this study, the connection between *NR1H3* and *NR0B2* was significant (> 0.7). Thus, the *NR1H3* transcription factor as well as *NR0B2* may have functional capacity to affect glucocorticoid receptors and impact their downstream effects and therefore warrant further evaluation for their roles as potential robust biomarkers for the selection of cattle with improved feed efficiency status.

## Conclusions

Results from this study highlight the interaction of genes related to divergence of the RFI phenotype in contrasting breeds of cattle offered varying diets. Biological functions related to immune function, lipid metabolism and energy production were implicated towards variation in RFI across varying breeds, diet types and tissues examined. Although no single gene was identified across the various contrasts and tissues examined, within the liver tissue, *IRX4*, *NR1H3*, *HOXA13* and *ZNF648* gene nodes displayed significant connections across the RFI, diet and breed comparisons, indicating a role for these genes towards the RFI phenotype irrespective of varying dietary source and breed type. Moreover, the *NR1H3* gene encodes a protein secreted into plasma from the hepatocytes of the liver, highlighting potential for this gene to be explored as a robust biomarker for the RFI trait in beef cattle.

## Methods

This study was conducted at the Teagasc Animal & Grassland Research and Innovation Centre Grange, Co. Meath in Ireland. All procedures involving animals were reviewed and approved by the Teagasc Animal Ethics Committee and all procedures involving animals were conducted under an experimental license (AE19132/P029) issued by the Health Products Regulatory Authority in Ireland in accordance with the cruelty to Animals Act 1876 and the European Communities (Amendment of Cruelty to Animals Act 1876) Regulations 2002 and 2005. All experiments were performed in accordance with relevant regulations and the ARRIVE (Animal Research: Reporting on In Vivo Experiments) guidelines.

### Animal management and phenotype collection

**Fig. 6:**
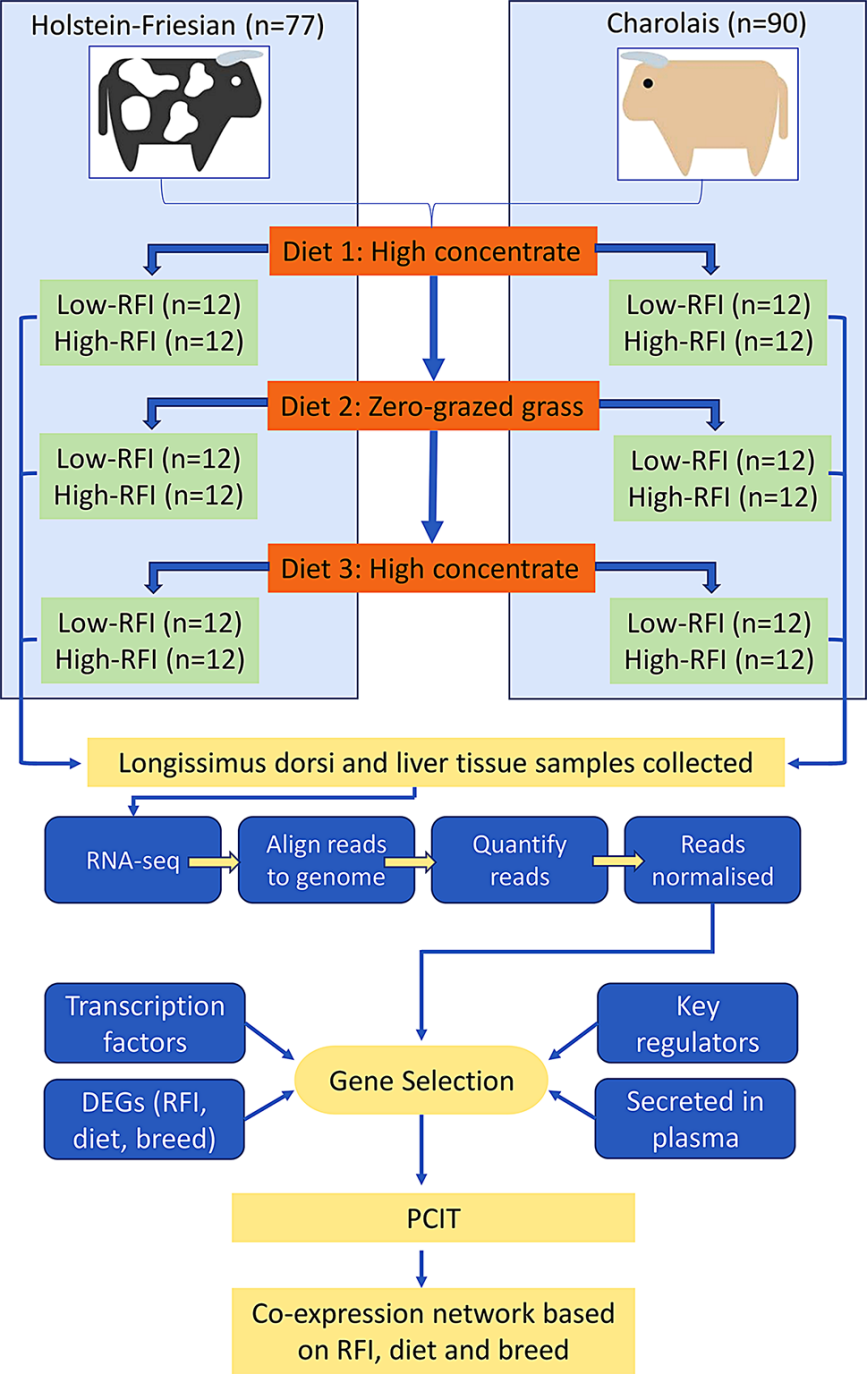
Overview of experimental design

This experiment was conducted as part of a larger research programme designed to examine the within-animal repeatability of feed intake, growth and feed efficiency between varying stages of development in Charolais and Holstein-Friesian beef steers offered contrasting diets over consecutive test periods [31, 32]. An overview of the experiment conducted in the current study is presented in Fig. 6. In total, 167 steers, comprised of 90 Charolais and 77 Holstein-Friesian were sourced for use in this study. Contrasting dietary phases consisted of (i) a high-concentrate diet during the growing phase (H1); (ii) zero-grazed grass diet during the growing phase (ZG) and; (iii) high-concentrate diet during the finishing phase (H2). At the start of the trial mean bodyweight (SD) and age (SD) were 485 kg (38.0) and 373 days (18.0) for Charolais steers, and 401 kg (43.3) and 399 days (7.6) for the Holstein-Friesian steers. Following a 14-day dietary adaptation period, individual animal intake and growth measurements were recorded over the three 70-day feeding phases, using an electronic Calan gate system (American Calan Inc., Northwood, NH, USA). All steers were weighed at the beginning and end of each dietary phase as well as on a fortnightly basis throughout. During the two high-concentrate phases, steers were offered the same concentrate *ad libitum* (86% rolled barley, 6% soya bean meal, 6% molasses, and 2% minerals and vitamins) with a restricted daily allowance of grass silage. For the zero-grazed grass phase, steers were individually offered fresh herbage, harvested twice daily from *Lolium perenne* dominant swards, *ad libitum*. Chemical composition of diets offered is detailed in full in Higgins et al. [10]. All steers had unrestricted access to fresh, clean drinking water throughout the trial.

Full details related to the computation of RFI trait are described in Higgins et al. [10]. Briefly, the residuals of the regression of dry matter intake (DMI) data on average daily gain (ADG) and mid-test metabolic BW (MBW) within each breed, were used to compute individual animal RFI coefficients for each feeding phase using the GLM procedure of SAS 9.4 (SAS Inst. INC., Cary, NC). Residual feed intake was calculated for each animal as the difference between actual DMI and expected DMI. At the end of each dietary phase, within breed, steers were ranked for having high-RFI (feed-inefficient; *n* = 12) and low-RFI (feed-efficient; *n* = 12) and were subsequently used for tissue sample collection.

### Tissue sample collection and RNA-Sequencing

Tissue sample collection (*n* = 12 for each High and Low-RFI groups) for liver and skeletal muscle are described in Higgins et al. [10] and Keogh et al. [12], respectively. Briefly, liver tissue was harvested from selected steers divergent for RFI at the end of each dietary phase by percutaneous punch between the 11th and 12th ribs as previously described by McCarthy et al. [33]. *Longissimus dorsi* muscle tissue samples were collected from the same animals as per the liver biopsy through punch biopsy between the 12th and 13th ribs. For both liver and muscle biopsies all steers received local anaesthetic (5 ml; Adrenacaine, Norbrook Laboratories, Ireland Ltd.) prior to biopsy collection. All instruments used for biopsy collection were sterilised, washed with 70% ethanol and treated with RNaseZap (Ambion, Applera Ireland, Dublin, Ireland), prior to use. For both liver and muscle biopsies, care was taken to ensure that samples were consistently harvested from the same location from each animal. Following collection, all tissue samples were washed with sterile DPBS and immediately snap frozen in liquid nitrogen before subsequent storage at -80 °C pending further processing.

For both liver and muscle biopsies, 50 mg of tissue sample was used for the isolation of total RNA. Tissue samples were homogenised in 3 ml of QIAzol reagent using a rotor-stator tissue lyser (Qiagen, UK). RNA was subsequently precipitated and purified using the RNeasy plus Universal kit (Qiagen, UK) according to the manufacturer’s instructions. The resultant quantity of total RNA isolated was determined by measuring the absorbance at 260 nm using a Nanodrop spectrophotometer (Nanodrop Technologies, Wilmington, DE, USA). RNA quality was determined using the RNA 6000 Nano Lab Chip kit (Aglient Technologies Ireland Ltd., Dublin, Ireland) on an Aglient Bioanalyser 2100. Adequate RNA quality was determined by ensuring that all RNA samples had a RIN (RNA integrity number) greater than 8. All RNA samples displayed RINs greater than 8 and thus were of suitable quality for subsequent RNA-sequencing. Individual cDNA libraries were prepared from each separate liver and muscle RNA sample for cattle divergent for RFI across each breed and dietary phase. cDNA libraries were prepared using the Illumina TruSeq stranded mRNA sample prep kit (Illumina, San Diego, CA, USA) according to the manufacturer’s instructions. Resultant cDNA libraries were validated using the DNA 1000 Nano Lab Chip kit on the Aglient Bioanalyser 2100. Sequencing was subsequently undertaken on an Illumina HiSeq 2500 sequencer. All sequencing data used in this study are publicly available in NCBI’s Gene Expression Omnibus and can be accessed through GEO IDs GSE113135 and GSE111464.

### RNAseq data analysis

Bioinformatic analysis was undertaken as previously described in Higgins et al. [10]. Briefly, quality control of sequencing reads was undertaken using FastQC (v 0.11.5; [34]), followed by removal of sequencing adapters and any low quality reads using Cutadapt (v 1.13; [35]). Trimmed sequencing reads were mapped to the bovine reference genome (ARS-UCD1.2; [36]) and also quantified using STAR (v.2.5.1; [37]). Differential expression was undertaken using the edgeR package within the R environment [38]. Due to issues related to poor RNA, or sequencing read quality some samples were removed from the analysis. This resulted in the following sample size for differential expression analysis in muscle: H1 diet: Charolais Low-RFI *n* = 8; Charolais High-RFI *n* = 10; Holstein-Friesian Low-RFI *n* = 9; Holstein-Friesian High-RFI *n* = 10; ZG diet: Charolais Low-RFI *n* = 9; Charolais High-RFI *n* = 8; Holstein-Friesian Low-RFI *n* = 9; Holstein-Friesian High-RFI *n* = 10; H2 diet: Charolais Low-RFI *n* = 8; Charolais High-RFI *n* = 8; Holstein-Friesian Low-RFI *n* = 8; Holstein-Friesian High-RFI *n* = 8. For the liver tissue, the following sample size was used for differential expression analysis: H1 diet: Charolais Low-RFI *n* = 10; Charolais High-RFI *n* = 11; Holstein-Friesian Low-RFI *n* = 10; Holstein-Friesian High-RFI *n* = 10; ZG diet: Charolais Low-RFI *n* = 8; Charolais High-RFI *n* = 9; Holstein-Friesian Low-RFI *n* = 9; Holstein-Friesian High-RFI *n* = 9; H2 diet: Charolais Low-RFI *n* = 10; Charolais High-RFI *n* = 11; Holstein-Friesian Low-RFI *n* = 8; Holstein-Friesian High-RFI *n* = 10. Specific animals used for each breed and dietary time-point are detailed in full in Additional Table 3. Within edgeR, gene expression reads were estimated as Counts Per Million (CPM) and genes which presented with at least 1 CPM in at least half of the samples were retained for differential expression analysis. Differentially expressed genes within each tissue were identified for each of the main contrasts of RFI phenotype (Low-RFI versus High-RFI), breed (Charolais versus Holstein-Friesian) and dietary source (HC versus ZG). Contrasts for DEGs were undertaken in edgeR for each tissue type separately, using a generalised linear model in each instance with DEGs determined through likelihood ratio testing. For the diet contrast, time-point was included within the model in order to account for the variation in timing for the two high concentrate dietary phases. The Benjamini-Hochberg correction [39] was applied to all p-values from differential expression contrasts undertaken.

### Co-expression network analysis

From the differential expression analysis conducted in edgeR, the top 5% DEGs, based on corrected p-value were selected for subsequent inclusion in the co-expression network analysis. The top 5% of DEGs was selected as a random nominal cut-off for the selection of genes that were most relevant to each specific contrast for subsequent inclusion in the co-expression network analysis. Additionally, key regulatory genes and proteins secreted in plasma were also utilised for network analysis. Specifically, the human secretome database [40] was used to select genes encoding proteins secreted in plasma by either liver or muscle tissues. Key regulatory genes were obtained from the Animal Transcription Factor Database [41]. The 1,445 known bovine transcription factors were evaluated for their regulatory potential by testing their expression profile against the DEGs for each contrast across each tissue type. This analysis was based on the Regulatory Impact Factor metrics (RIF; [42]), which is comprised of a set of two metrics whereby scores are assigned to regulatory genes that are consistently differentially co-expressed with target genes (RIF1) and to those with the greatest altered ability to predict the abundance of their target genes were considered significant (RIF2). Genes were selected for co-expression network analysis if (i) differentially expressed, (ii) transcription factor, including key transcription factors resulting from the RIF analysis; and (iii) secreted in plasma. Significant connections (edges) between nodes were identified using the Partial Correlation and Information Theory (PCIT) algorithm [43], which determines the significance of the correlation between a pair of nodes (genes) after accounting for all other nodes within the network. Connections between gene nodes were accepted when the partial correlation was greater than two standard deviations from the mean (*P* < 0.01). The resultant network of co-expressed genes was imported into Cytoscape software [44] for visualisation. Highly interconnected regions or clusters of co-expressed genes were identified through the MCODE application within Cytoscape [45]. In order to assign biological annotation of the generated network, clusters of highly interconnected genes were further evaluated for functional enrichment using Ingenuity Pathway Analysis (IPA, Qiagen, [46]).

Additionally, the relationship between genes within each of the RFI, breed and diet contrasts in each tissue with regulatory genes was also assessed. This was achieved through firstly identifying the most interconnected gene within each of the main contrasts (RFI, breed and diet) across each of the two tissue types. A second network was then depicted determining the first neighbours of the most interconnected genes that are transcription factors, determined from the Animal Transcription Factor Database [41]. The resultant network was visualised using Cytoscape software [44].

### Electronic supplementary material

Below is the link to the electronic supplementary material.

Supplementary Material 1

Supplementary Material 2

Supplementary Material 3

## Data Availability

The transcriptomic datasets utilised for this study can be found in the NCBI’s Gene Expression Omnibus (GEO) database [https://www.ncbi.nlm.nih.gov/geo/] (GEO accession IDs: GSE113135 and GSE111464).

## Abbreviations

ADG: average daily gain
CPM: counts per millions
DEG: differentially expressed gene
DMI: dry matter intake
DPBS: Dulbecco’s phosphate buffered saline
GEO: gene expression omnibus
H1: high concentrate diet during the growing phase
H2: high concentrate diet during the finishing phase
HC: high concentrate
IPA: ingenuity pathway analysis
MBW: mid-test metabolic weight
RFI: residual feed intake
RIF: regulatory impact factor
RIN: RNA integrity number
ZG: zero-grazed grass diet during the growing phase
