# Supplementary figures and images for: An across breed, diet and tissue analysis reveals the transcription factor NR1H3 as a key mediator of residual feed intake in beef cattle
Source: BMC Genomics. 2024 Mar 4;25:234. doi: 10.1186/s12864-024-10151-2 (PMC10910725; doi:10.1186/s12864-024-10151-2)

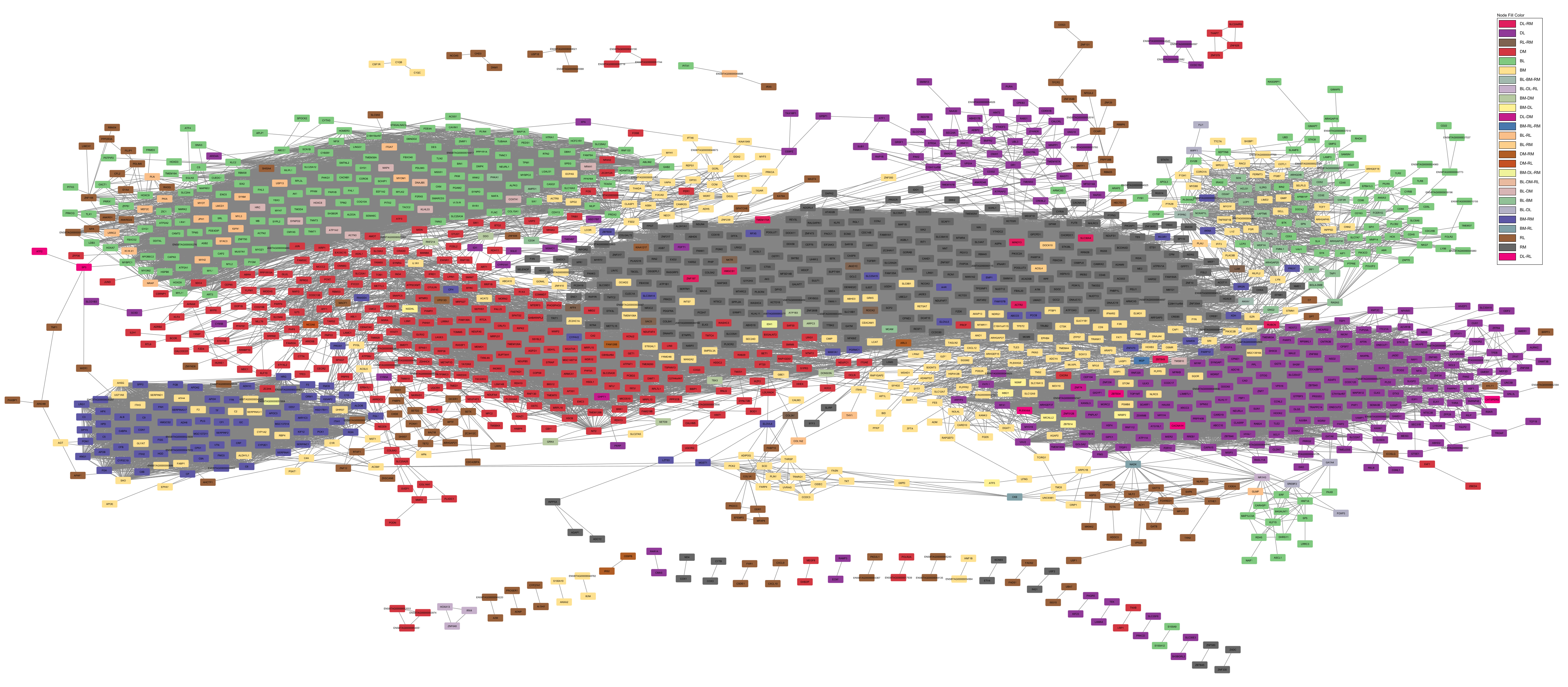

Supplement: Supplementary file 1 — Supplementary Material 1 [file 12864_2024_10151_MOESM1_ESM.tif]

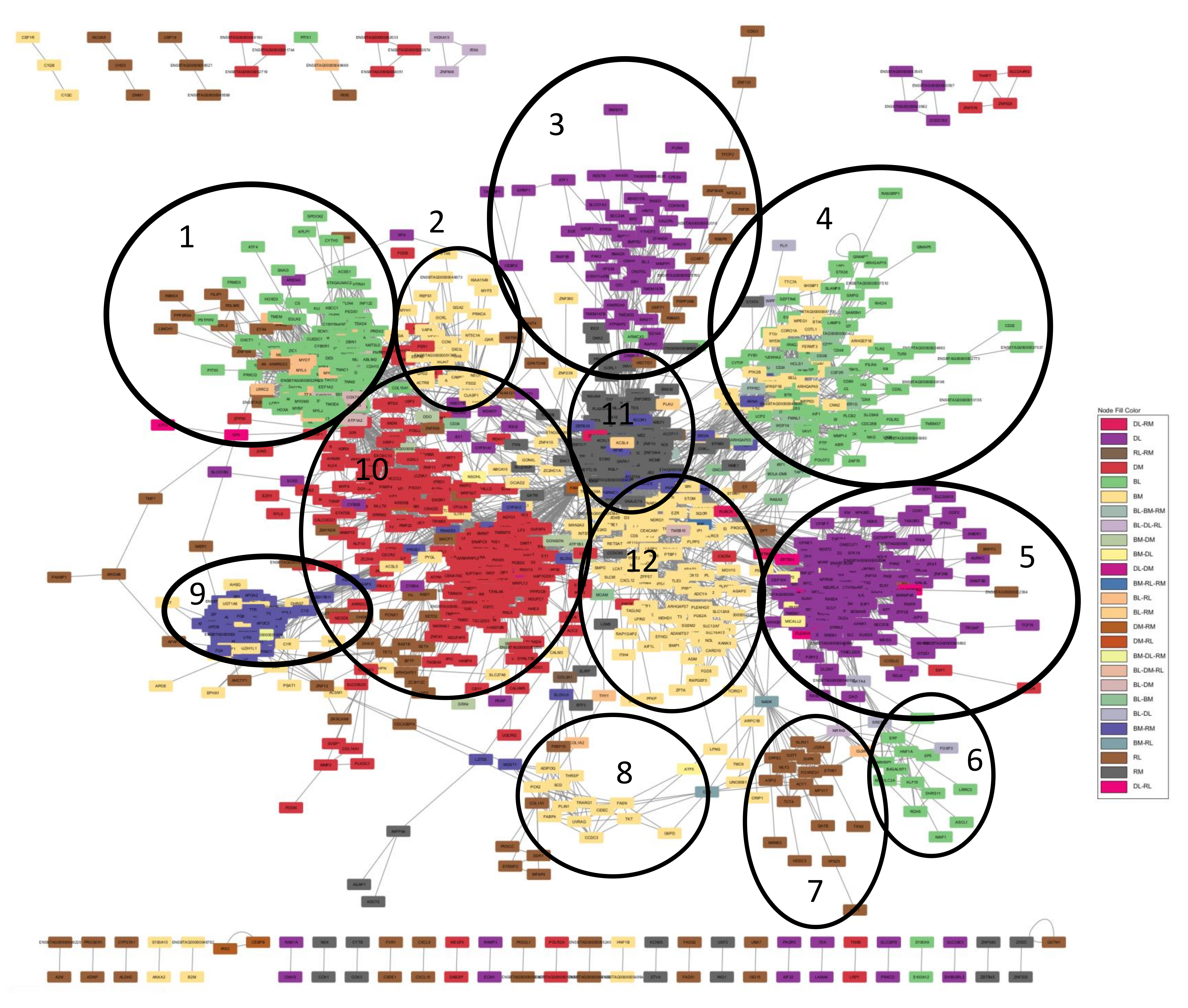

Supplement: Supplementary file 2 — Supplementary Material 2 [file 12864_2024_10151_MOESM2_ESM.tif]
